# RegX3-Mediated Regulation of Methylcitrate Cycle in *Mycobacterium smegmatis*

**DOI:** 10.3389/fmicb.2021.619387

**Published:** 2021-02-02

**Authors:** Jin-Feng Pei, Nan Qi, Yu-Xin Li, Jing Wo, Bang-Ce Ye

**Affiliations:** ^1^Institute of Engineering Biology and Health, Collaborative Innovation Center of Yangtze River Delta Region Green Pharmaceuticals, College of Pharmaceutical Sciences, Zhejiang University of Technology, Hangzhou, China; ^2^Lab of Biosystems and Microanalysis, State Key Laboratory of Bioreactor Engineering, East China University of Science and Technology, Shanghai, China

**Keywords:** *Mycobacterium smegmatis*, phosphate metabolism, regX3 regulator, methylcitrate cycle, propionate metabolism

## Abstract

*Mycobacterium tuberculosis* is a global human pathogen that infects macrophages and can establish a latent infection. Emerging evidence has established the nutrients metabolism as a key point to study the pathogenesis of *M. tuberculosis* and host immunity. It was reported that fatty acids and cholesterol are the major nutrient sources of *M. tuberculosis* in the period of infection. However, the mechanism by which *M. tuberculosis* utilizes lipids for maintaining life activities in nutrient-deficiency macrophages is poorly understood. *Mycobacterium smegmatis* is fast-growing and generally used to study its pathogenic counterpart, *M. tuberculosis*. In this work, we found that the phosphate sensing regulator RegX3 of *M. smegmatis* is required for its growing on propionate and surviving in macrophages. We further demonstrated that the expression of *prpR* and related genes (*prpDBC*) in methylcitrate cycle could be enhanced by RegX3 in response to the phosphate-starvation condition. The binding sites of the promoter region of *prpR* for RegX3 and PrpR were investigated. In addition, cell morphology assay showed that RegX3 is responsible for cell morphological elongation, thus promoting the proliferation and survival of *M. smegmatis* in macrophages. Taken together, our findings revealed a novel transcriptional regulation mechanism of RegX3 on propionate metabolism, and uncovered that the nutrients-sensing regulatory system puts bacteria at metabolic steady state by altering cell morphology. More importantly, since we observed that *M. tuberculosis* RegX3 also binds to the *prpR* operon *in vitro*, the RegX3-mediated regulation might be general in *M. tuberculosis* and other mycobacteria for nutrient sensing and environmental adaptation.

## Introduction

Tuberculosis, a high lethality illness induced by *Mycobacterium tuberculosis* (*M. tuberculosis*), is responsible for millions of deaths every year. As extremely successful intracellular pathogen, *M. tuberculosis* exerts a latent infection in macrophages by a series of adaptive mechanisms. These strategies enable *M. tuberculosis* to quickly adapt to environmental changes, including the acidic conditions (pH 5.8-6.2), the nutrient depleted and hypoxic center of granulomas (Sturgill-Koszycki et al., 1994; Russell et al., 2010; Flynn et al., 2011). Notably, two-component regulatory systems (TCRSs) are the major adaptive mechanisms that affect global regulation in response to the specific environmental stimulus, thereby contributing to the long-term survival of *M. tuberculosis* within host cells (Rickman et al., 2004). Genomic analysis of *M. tuberculosis* has identified eleven kinds of TCRSs as well as several unpaired HKs and RRs. It was reported that some of these components are crucial for the adaptive responses under stress conditions and the pathogen virulence of *M. tuberculosis* (James et al., 2012).

SenX3-RegX3 system is the first identified TCRS in *M. tuberculosis* (Wren et al., 1992). Like other highly conserved TCRS, Mycobacteria SenX3-RegX3 is an auto-regulation operon and consist of a sensor (senX3) and its regulator regX3 (Himpens et al., 2000). When sensor protein detects environmental signals, the conserved histidine is self-phosphorylated and then transfers a phosphoryl group to response regulator to activate its DNA-binding capability, thus leading to the induction or suppression of target genes (Stock and Guhaniyogi, 2006). Interestingly, the intergenic region (IR) between *senX3* and *regX3* consists of several mycobacterial interspersed repetitive units (MIRUs), which may not only participate in regulation of gene expression by stabilizing upstream mRNA and controlling translation of downstream genes within a polycistronic operon, but also modulate host–pathogen interactions (Rifat and Karakousis, 2014). It was found that *M. tuberculosis senX3*-*regX3* null mutant in infected macrophages and a mice model showed an apparent decline. Consequently, SenX3-RegX3 system is essential for *M. tuberculosis* virulence, and also indispensable for bacillary survival in host cells and lungs (Parish et al., 2003; Rickman et al., 2004; Rifat et al., 2009). More importantly, SenX3-RegX3 is a regulator of the genes referred to phosphate uptake in Mycobacterium. In this system, the dual roles of SenX3 as a phosphatase or phosphate donor of response regulator RegX3 depend on phosphate availability, while inorganic phosphate assimilation, regulated by the inorganic phosphate concentration within environment, is required for Mycobacterium growth and infection (Glover et al., 2007; Guo et al., 2009; Tischler et al., 2013). It is noticed that *senX3*-*regX3* expression is not activated when dealing with a wide range of stress conditions like extremes of the pH scale, antibiotics and hydrogen peroxide, but is significantly increased in the case of low phosphorus. Therefore, SenX3-RegX3 controls the genes expression of Phosphate dose-dependence in Mycobacterium and is vital for optimum growth under phosphate-restricted condition (Glover et al., 2007). Moreover, it was reported that *M. tuberculosis* relies on a Pst system to regulate RegX3 activity in response to the available phosphate *in vivo*. The inorganic phosphate, a common phosphate source for bacteria to replicate in lungs, is a crucial signal regulating the *M. tuberculosis* gene expression through the Pst-SenX3-RegX3 signal transduction system (Tischler et al., 2013). Microarray analysis showed that SenX3-RegX3 regulated nearly 100 genes in *M. tuberculosis*, which were related to large numbers of important processes, including biosynthesis of biological macromolecules (DNA and RNA), oxidative stress, synthesis of PE/PPE family proteins, cell wall synthesis, fatty acid degradation and toxicity (Guo et al., 2009). The observation indicated that RegX3 might be a potential global regulator in mycobacteria.

Emerging evidence has demonstrated that *M. tuberculosis* utilizes fatty acids and cholesterol as main carbon sources during infection (Bloch and Segal, 1956; Mckinney et al., 2000; Schnappinger et al., 2003; Lee et al., 2017). In Mycobacterium metabolism, fatty acids enter the bacteria via membrane free diffusion and relevant transporters, and then go into β-oxidation cycle for gradual breakdown (Munoz-Elias and McKinney, 2006). Through continuous cycles of β-oxidation, even-chain and odd-chain fatty acids were converted into acetyl-coenzyme A (acetyl-CoA) and propionyl-coenzyme A (propionyl-CoA), which could be further oxidized through glyoxylate shunt and methylcitrate cycle, respectively (Rhee et al., 2011; Anthony and Kyu, 2014). It should be emphasized that methylcitrate cycle makes great contribution to the Mycobacterium growth in propionate and odd-chain fatty acids, since the propionate accumulated in environment and the propionate metabolites generated by β-oxidation are toxic, thus significantly inhibiting bacterial growth. The methylcitrate cycle can convert propionate into propionyl-CoA, which is subsequently oxidized into pyruvate by three specific enzymes, 2-methylisocitrate lyase (PrpB), methylcitrate synthase (PrpC) and methylcitrate dehydratase (PrpD) (Munoz-Elias and McKinney, 2006; Upton and McKinney, 2007; Puckett et al., 2017). Moreover, a novel transcription factor PrpR has been first described by Datta et al. and is important for the metabolism of odd-chain fatty acids and cholesterol in *M. tuberculosis* (Datta et al., 2011). PrpR can directly regulate the encoding genes for methylcitrate (*prpC*, *prpD*) and glyoxylate (isocitrate lyase, Icl1) circulations (Masiewicz et al., 2012). When utilizing propionate as the sole carbon source, the *prpR*-deletion strain of *M. tuberculosis* exhibited a compromised growth *in vitro*. PrpR activates the transcription of *prpDC* and *icl1* genes, but also negatively regulates *ramB*, the *prpR* homolog controlling glyoxylate cycle. Furthermore, it has been underlined that *M. tuberculosis prpR* is crucial for the bacterial growth and survival in hypoxic hollow fiber model in mice (Klinkenberg et al., 2008).

The current studies about RegX3 mainly focus on its regulation of phosphorus metabolism in *M. tuberculosis*. However, the regulation effects of RegX3 on other physiological processes were poorly understood. A previous study uncovered a regulatory role of RegX3 in the expression of *prpC* in *M. tuberculosis*, suggesting that RegX3 might regulate the methylcitrate cycle (Roberts et al., 2011). In this work, we expanded the research scope of RegX3 regulation and explored its influence on carbon metabolism route. Due to the high pathogenicity and long growth cycle of *M. tuberculosis*, we selected its fast-growing counterpart *Mycobacterium smegmatis* (*M. smegmatis*) as the experimental object. *M. smegmatis*, a common model used for rapid screening of new antituberculotic drugs, is an atypical mycobacterium with high-speed proliferation and no pathogenicity (Sweeney et al., 2011). Using this model, we found that RegX3 is necessary for the *M. smegmatis* survival and growth in propionate. RegX3 directly up-regulated PrpR to promote the transcription of *prpC*, *prpD* and *prpB* encoding key enzymes of methylcitrate, thereby activating the whole methylcitrate cycle. This regulation mode also responds to the environmental phosphorus concentration and relies on the phosphorus availability. Furthermore, it was indicated that RegX3 promoted *M. smegmatis* proliferation and survival by changing the strain morphology in macrophages. Our data thus establishes a comprehensive regulatory network mediated by RegX3, and reveals the metabolic status of fatty acid intermediate metabolites in *M. smegmatis*.

## Results

### RegX3 Promotes the Growth of *M. smegmatis* on Propionate

**FIGURE 1:**
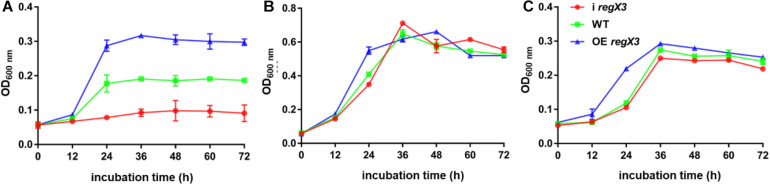
Growth curves of diverse *M. smegmatis* strains grown under different conditions. The three *M. smegmatis*, WT, i *regX3*, and OE *regX3*, were cultured in panel **(A)** MOPS-propionate (10 mM) minimal medium with low concentration phosphate (100 μM K_2_HPO_4_), **(B)** MOPS-glucose (10 mM) minimal medium with a low concentration of phosphate (100 μM K_2_HPO_4_) and **(C)** MOPS-propionate (10 mM) minimal medium with a high concentration of phosphate (10 mM K_2_HPO_4_), respectively. The mean of OD_600_ nm was calculated at each time point. Data are presented as means ± SEM calculated from three independent experiments.

To investigate the role of the *MSMEG_0937*-encoded RegX3 in *M. smegmatis* propionate metabolism, we constructed a *regX3* knock-down strain by the CRISPR interference (CRISPRi)-based genetic repression, as well as a *regX3* overexpression strain. Changes in *regX3* transcription levels of the engineered stains were measured (Supplementary Figure 1). The growth curves of *M. smegmatis* wild-type (WT), the knock-down strain (i *regX3*) and the overexpression strain (OE *regX3*) in MOPS-propionate (10 mM) minimal medium with 100 μM K_2_HPO_4_ as its phosphorus source were measured. As shown in Figure 1A, the differences were observed among the growth of three strains. Compared to WT, the biomass of OE *regX3* increased to a nearly 1.5-fold after 24 h, while i *regX3* hardly grew on propionate. However, the three strains of *M. smegmatis* exhibited similar growth curves when they were cultured in 10 mM glucose (as sole carbon source) or 10 mM K_2_HPO_4_ (as higher phosphate condition) (Figures 1B,C). These data indicated an essential role of RegX3 in the propionate catabolism, which is consistent with the previous findings that RegX3 is involved in the regulation of fatty acid metabolism (Parish et al., 2003).

### *Mycobacterium smegmatis* RegX3 Binds to the Promoter Region of *prpR* Gene

**FIGURE 2:**
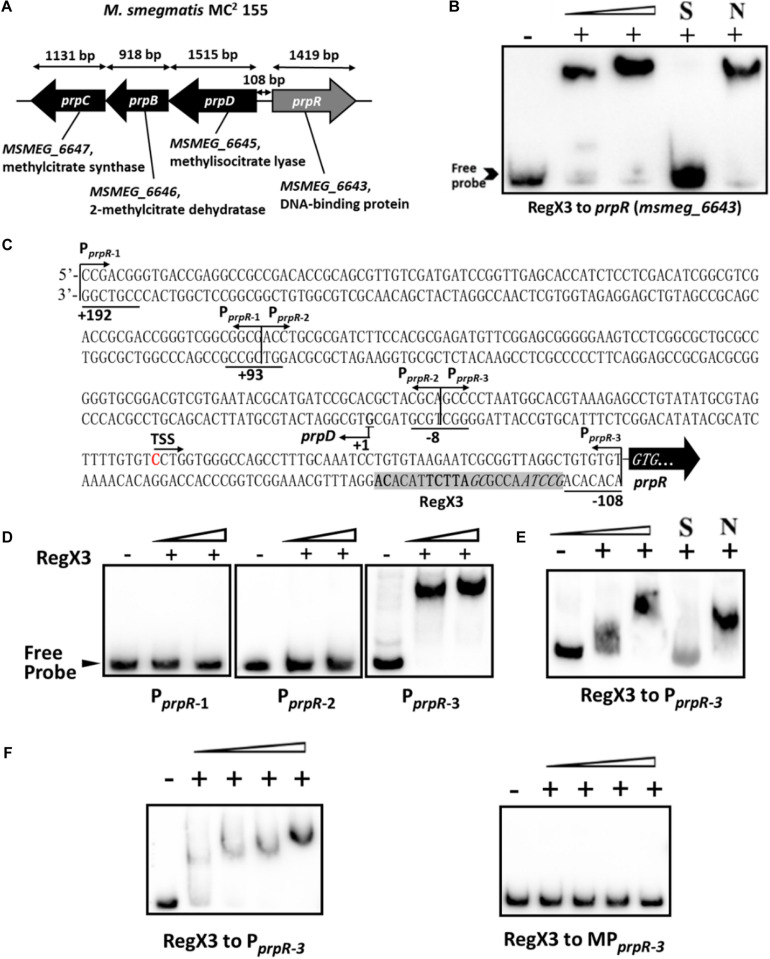
Binding of RegX3 to the regulatory region of the genes related to the methylcitrate cycle. **(A)** The schematic depiction of *prpDBC* (*msmeg_6645-6647*) and *prpR* (*msmeg_6643*) regions on the *M. smegmatis* MC^2^ 155 chromosome. The open reading frame *prpR* is located in close proximity to the *prpDBC* operon. **(B)** EMSA showing the binding of His-RegX3 with the promoter of *prpR*. The biotin-labeled probe, a 300-bp DNA fragment corresponding to the upstream region (-300 to 0 bp) of *prpR*, was co-incubated with different concentrations of recombinant His-RegX3 protein (0, 0.6, and 1.2 μM). S represents the unlabeled specific probe, N represents the non-specific competitor DNA (Salmon sperm DNA). **(C)** Putative binding site (shadowed) of RegX3 in the promoter regions of *prpR*. The +192, +93, -8, and -108 positions are underlined; the three short fragments (P*_*prpR–1*_*, P*_*prpR–2*_*, and P*_*prpR–3*_*) and TSS (transcriptional start site) are marked. **(D)** EMSA showing the binding of His-RegX3 (0, 3.6, and 7.1 μM) with three biotin-labeled short fragments from *prpR* upstream promoter region. **(E)** Binding of biotin labeled P*_*prpR–3*_* with increasing concentration of His-RegX3 (0, 1.6, and 3.2 μM). **(F)** EMSA showing the binding of His-RegX3 (0, 0.35, 0.7, 1.4, and 2.8 μM) with biotin-labeled P*_*prpR–3*_* containing predicted consensus binding motif (GCCTAACCGCGATTCTTACACA) (left) or biotin-labeled MP*_*prpR–3*_* containing mutate binding motif (**T**CCT**C**ACCGCGA**GGA**TTACACA) (right).

Next, we sought to explore the underlying mechanism by which RegX3 promotes *M. smegmatis* growth on propionate. Electrophoretic mobility shift assay (EMSA) showed that recombinant RegX3 of *M. smegmatis* specifically bound to the upstream promoter region of *prpR* (*MSMEG_6643*) gene *in vitro* (Figures 2A,B). Since PrpR is a transcription factor referred to the regulation of methylcitrate route in *M. smegmatis* (Masiewicz et al., 2012), we suspected that RegX3 affects the whole methylcitrate cycle in *M. smegmatis* by regulating *prpR* gene. In addition, the binding of RegX3 to the promoter region of *prpR* gene in *M. tuberculosis* was validated (Supplementary Figure 2), indicating that the RegX3-mediated transcriptional regulation of methylcitrate cycle might be conserved in *M. tuberculosis*.

Previous studies have identified the DNA-binding site of RegX3 with a conservative binding sequence, GT/GTCAcc-n_4_-cG (Glover et al., 2007). Using MAST/MEME tools and PREDetector software, we identified an unknown putative RegX3 binding motif in the promoter region of *prpR* (Figure 2C, marked by shadow background). To precisely determine the RegX3 binding position in *prpR* operon, we generated three about 100 bp DNA fragments derived from the *prpR* gene upstream and promoter region by PCR, named P*_*prpR–*1_*, P*_*prpR–*2_*, and P*_*prpR–*3_*. As expected, P*_*prpR–*3_* harboring the predicted RegX3 binding site was bound to RegX3 (Figures 2D,E). To further confirm the specific binding, we introduced point mutations in the conserved sites of the binding motif. No shift band was observed in EMSA assays for MP*_*prpR–*3_*, a biotin-labeled probe derived from P*_*prpR–*3_* with mutant motif (Figure 2F). Altogether, our data characterized a previous unknown RegX3-binding motif in the promoter region of *prpR* in *M. smegmatis* and *M. tuberculosis*.

### Induction of the Methylcitrate Cycle-Related Genes in Response to Phosphate Starvation

**FIGURE 3:**
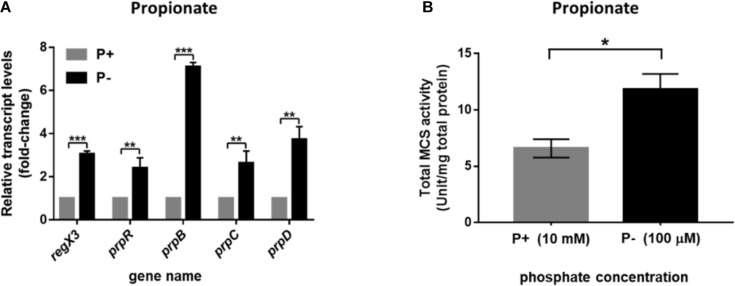
Induction of the methylcitrate cycle-related genes and enzyme activity in *M. smegmatis* in response to different phosphate availabilities. **(A)** The transcriptional profiles of *regX3* and methylcitrate-cycle-related genes when *M. smegmatis* was grown in MOPS-propionate (10 mM) medium without phosphate (P-) or with a high phosphate level (P+, 10 mM K_2_HPO_4_). RNA was collected at 12 h after incubation. Relative fold change was tested using 16S rRNA as housekeeping gene. The data of relative fold change under the high phosphate condition was set to 1.0 (arbitrary unit). **(B)** Comparison analysis of total MCS activity in *M. smegmatis* grown at the low- and high-phosphate level. Ten micrograms of total protein were used in the MCS activity assay. Data are presented as means ± SEM calculated from three independent experiments. Unpaired two-tailed Student’s *t*-test, *p < 0.05; **p < 0.01; and ***p < 0.001.

As previously reported, the phosphate acquisition in *M. smegmatis* is mediated by a two-component regulatory system SenX3-RegX3 in response to environmental inorganic phosphate concentration. We further investigated the transcriptional profile of operon *prpDBC* and the activity of MCS enzyme in *M. smegmatis* under the conditions of different phosphate availabilities. As described in section “Materials and Methods,” the *M. smegmatis* WT strain harvested at logarithmic phase in LB broth with 0.05% Tween 80 were transferred to modified MOPS-propionate (10 mM) minimal media (100 μM K_2_HPO_4_ of low-phosphate condition and 10 mM K_2_HPO_4_ of high-phosphate condition) for 12-h incubation. The significant increase of *regX3* transcription level (3.1-fold, *p* < 0.001) was observed responding to phosphate starvation, which induced expression of the genes participating in methylcitrate pathway, including *prpR* (2.4-fold, *p* < 0.01), *prpB* (7.1-fold, *p* < 0.001), *prpC* (2.6-fold, *p* < 0.01), and *prpD* (3.7-fold, *p* < 0.01) (Figure 3A). In addition, intracellular total activity of MCS enzyme was increased in phosphate-limited medium, approximately 1.8-fold, over that in phosphate-rich medium (Figure 3B). These results indicated that the expression of methylcitrate cycle genes can be induced in response to phosphate starvation.

### RegX3 Activates the Methylcitrate Cycle by Up-Regulating *prpR* Transcription During *M. smegmatis* Growth on Propionate

**FIGURE 4:**
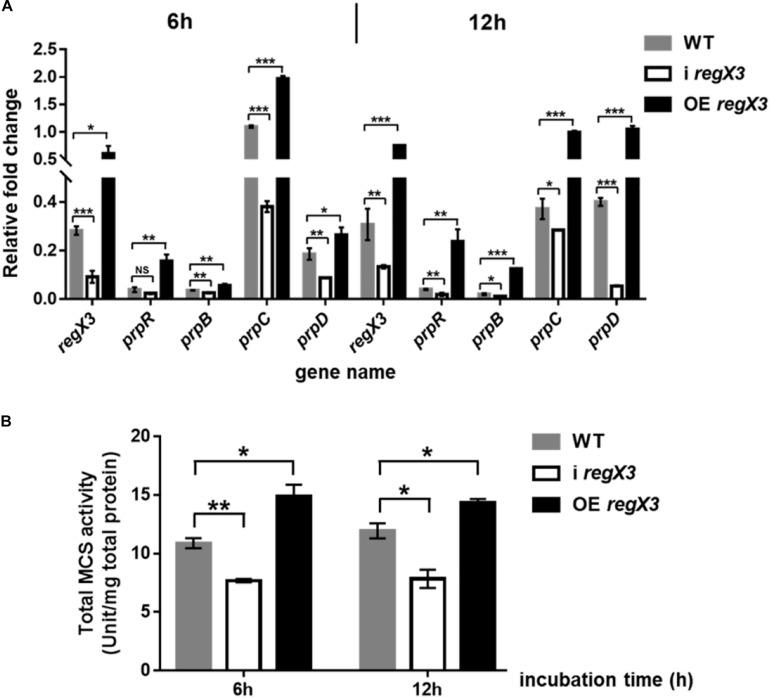
Induction of the methylcitrate cycle-related gene and enzyme activity by RegX3 in *M. smegmatis* growing on propionate under the low-phosphate condition. **(A)** Transcriptional profiles of *regX3* and the methylcitrate cycle-related genes in WT, i *regX3*, and OE *regX3* strains of *M. smegmatis* grown in MOPS-propionate (10 mM) medium supplemented with 100 μM K_2_HPO_4_. Cells were harvested for RNA extraction at 6 and 12 h after incubation. 16S rRNA was selected as a reference and fold change was analyzed by qRT-PCR. **(B)** Total activity of MCS enzyme in diverse *M. smegmatis* strains grown on propionate. Strains were incubated and collected under the same conditions as tested for the qRT-PCR analysis. 10 μg total protein were used. Data are presented as means ± SEM calculated from three independent experiments. Unpaired two-tailed Student’s *t*-test, *p < 0.05; **p < 0.01; and ***p < 0.001.

Methylcitrate cycle is the most widely studied pathway of propionyl-CoA metabolism in *M. smegmatis* (Munoz-Elias and McKinney, 2006; Munoz-Elias et al., 2006). Besides, it was reported that when propionate is the main carbon source, PrpR acted as a transcriptional activator of *prpD, prpB, prpC* encoding three key enzymes in methylcitrate pathway (Masiewicz et al., 2012). To investigate the effect of RegX3 on the methylcitrate cycle in *M. smegmatis*, we determined transcriptional levels of gene *prpR* and operon *prpDBC* and enzyme activity of MCS in the three strains (WT, i *regX3* and OE *regX3*) of *M. smegmatis*. Under the limiting culture conditions, diverse *M. smegmatis* strains were collected at 6- and 12-h post incubation. Compared to WT, i *regX3* resulted in down-regulation of methylcitrate cycle-related genes; while overexpression of *regX3* remarkably induced the expression of these genes (Figure 4A), indicating that RegX3 was a transcriptional activator of *prpR* gene. However, the impacts of RegX3 on the transcription of methylcitrate cycle related genes were disappeared when the three *M. smegmatis* strains grew on glucose (Supplementary Figure 3), indicating that the RegX3-mediated regulation of the methylcitrate cycle at low phosphate is propionate-dependent.

Next, we examined if RegX3 affects the activity of MCS, the key enzyme required for the first step that propionyl-CoA enters methylcitrate cycle. Consistent with the positive effects at transcriptional level, the transcription inhibition of *regX3* resulted in obvious decreases in total MCS activities compared to WT. On the contrary, overexpression of *regX3* enhanced MCS activities to about 1.4-fold (*p* < 0.05) and 1.2-fold (*p* < 0.05) after 6- and 12-h incubation, respectively (Figure 4B). These results demonstrated that RegX3 activates the methylcitrate cycle of *M. smegmatis* growing on propionate in response to phosphate starvation, through up-regulating the *prpR* transcription.

### RegX3 Competes With PrpR for Binding to the *prpR* Promoter

**FIGURE 5:**
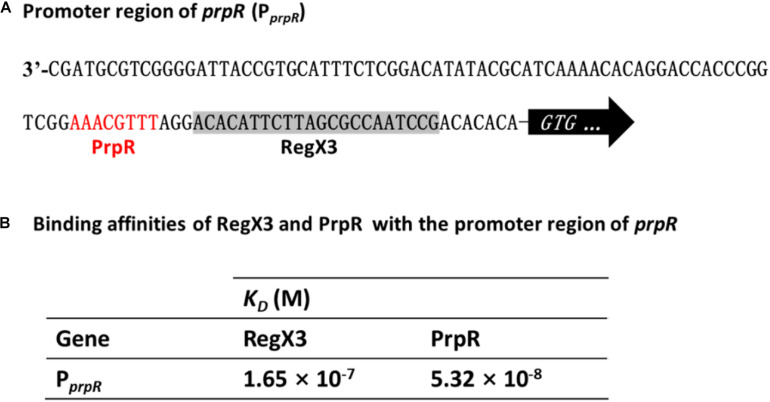
Competitive binding of RegX3 and PrpR to the *prpR* promoter. **(A)** Overview of P*_*prpR*_* showing the RegX3 box (shaded) and PrpR binding site (red). **(B)** Binding affinities of the promoter region of *prpR* for RegX3 and PrpR. The interactions of recombinant His-RegX3 and His-PrpR with the *prpR* (encoding MCS) were determined using the Octet Macromolecular Interaction Platform. The KD value of RegX3 and PrpR was 165 and 53.2 nM, respectively, which demonstrates that the PrpR has a stronger binding capacity than RegX3.

Previous studies identified a highly conserved PrpR-binding palindromic sequence (TTTGCAAA) in the promoter region of *prpR*, resulting in autoregulation of the PrpR protein (Masiewicz et al., 2012). As described in Figure 2C, RegX3 bound to the *prpR* promoter within the upstream region of a 22-bp sequence, which is similar to the typical RegX3-binding box GT/GTCAcc-n4-cG. Indeed, PrpR and RegX3 binding sites are very close to each other with only three bases in space (Figure 5A), indicating a potential competition for the *prpR* promoter between RegX3 and PrpR.

To further investigate which regulator protein has higher affinity and plays a major role in the competitive regulation of *prpR* gene, the kinetic binding analysis of RegX3 and PrpR to the *prpR* promoter were conducted using Bio-layer Interferometry. As shown in Supplementary Figures 4, 5B, serial concentrations of recombinant RegX3 or PrpR were incubated with DNA probes, and the affinity constant *K*_*D*_ was calculated by the measurement of association and dissociation. As described in Figure 5B, PrpR (*K_*D*_* = 165 nM) had a 2-fold higher affinity for the *prpR* promoter than that of RegX3 (*K_*D*_* = 53.2 nM), suggesting that PrpR prevents RegX3 from binding to the putative RegX3-Box in the promoter of *prpR*.

### RegX3 Promotes the Proliferation and Survival of *M. smegmatis* in Macrophages

Considering that RegX3 is required for *M. tuberculosis* resistance to phosphate-limiting stress and indispensable for *M. tuberculosis* survival in humans (Gomez and McKinney, 2004), we reasoned that the RegX3 of *M. smegmatis* might be also essential for its survival in macrophage cells. To investigate the effect of RegX3 on survival of *M. smegmatis* in macrophage, THP-1 macrophage cells were co-cultured with *M. smegmatis* WT, i *regX3*, and OE *regX3* strains. Survival of these strains were tested at different time points (2, 12, 24, 36, and 48 h). All these bacteria underwent growth and clearance in macrophages.

**FIGURE 6:**
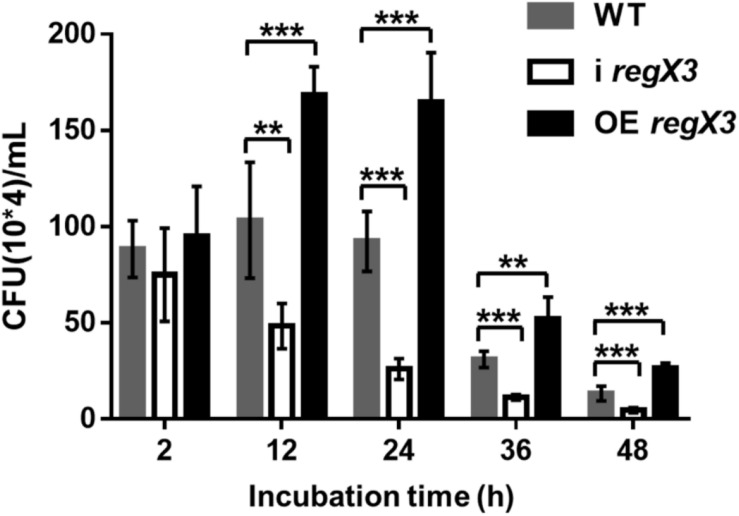
Survival of diverse *M. smegmatis* strains in macrophages. THP-1-derived macrophage cells were infected with *M. smegmatis* WT, i *regX3*, and OE *regX3* strains. Survival of these strains were determined in different time, 2, 12, 24, 36, and 48 h. Cells were harvested at different time points to determine the concentrations of live *M. smegmatis* (*n* = 6). Data are presented as means ± SEM calculated from three independent experiments. Unpaired two-tailed Student’s *t*-test, **p < 0.01 and ***p < 0.001.

As shown in Figure 6, the overexpression strain has a higher growth rate and a lower clearance rate during the macrophage infection period. No significant difference in the survival of the three strains in macrophages was observed at 2 h after infection. But the obvious differences appeared with the extension of infection time. The overexpression strain in macrophages doubled in the first 12 h of infection, and the CFU of bacteria at 12 h is almost twice of that at 2 h. At the same time, the WT strain had a slight increase, while the amount of i *regX3* decreased steadily. After 24 h of infection, the three strains in macrophages began to decline remarkably and maintained a stable difference in survival rate. At the 48 h time point, the CFU of OE *regX3* was about 2-fold (*p* < 0.001) higher than that of WT strain, while i *regX3* CFU was decreased approximately at one-half of WT CFU (*p* < 0.001). These results revealed that OE *regX3* has a higher growth rate and a lower clearance rate during infection of macrophage, suggesting that RegX3 exhibits a positive effect on the proliferation and survival of *M. smegmatis* in macrophages.

### RegX3 Affects the Cell Morphology of *M. smegmatis* Growing on Propionate

**FIGURE 7:**
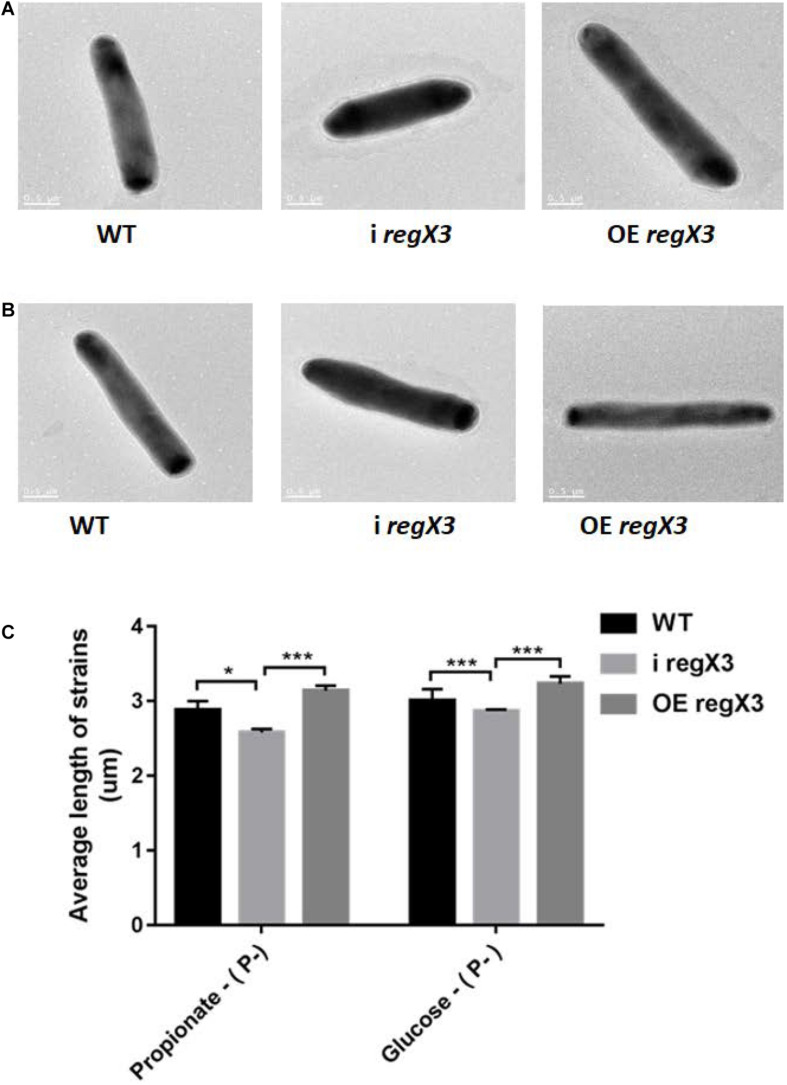
The morphology of three *M. smegmatis* strains growing on different carbon resources in response to phosphate starvation. Transmission electron microscopy (TEM) images of single bacillus from *M. smegmatis* WT, i *regX3*, and OE *regX3* strains after incubated for 12 h in the 10 mM MOPS-propionate **(A)**, or the 10 mM MOPS-glucose **(B)** minimal medium with a low concentration of phosphate (100 μM K_2_HPO_4_). Scale bar represents 0.5 μm. See also Supplementary Figures 5A,B. **(C)** Length of bacteria cells (*n* = 30) as shown in Figure S5A and S5B was measured by manual evaluation, using Image J on the electron microscopic images. Data are presented as means ± SEM. Unpaired two-tailed Student’s *t*-test, *p < 0.05 and ***p < 0.001.

It was previously reported that *M. tuberculosis* undergoes elongation following Pi depletion and this physiological change is closely related to the effect of SenX3-RegX3 (Rifat et al., 2009, 2014). To explore whether RegX3 also plays an essential role in the cell morphology of *M. smegmatis* growing on propionate, we measured the bacterial cell length of WT, i *regX3*, and OE *regX3* strains by using transmission electron microscopy (TEM). Images of thirty bacilli of each strain were randomly capture after cultivated for 12 h in the modified MOPS-propionate (10 mM) or the glucose (10 mM) minimal media supplemented with a low concentration of phosphate (100 μM K_2_HPO_4_) (Supplementary Figures 5A,B). As shown in Figures 7A–C, the mean length of i *regX3* (2.58 ± 0.05 μm) was shorter than that of either WT (2.88 ± 0.12 μm) or OE *regX3* (3.14 ± 0.07 μm) bacilli during growth on propionate in response to phosphate starvation. However, no significant difference was observed in the length of WT (2.84 ± 0.1 μm), i *regX3* (2.78 ± 0.05 μm), and OE *regX3* bacilli (2.93 ± 0.02 μm) cultured in glucose with a low concentration of phosphate. The morphological change related to *regX3* was disappeared when the three *M. smegmatis* strains sensed the high-phosphate condition (Supplementary Figures 5C–E). Collectively, these data indicated that RegX3 triggers morphological change of *M. smegmatis* growing on propionate in response to phosphate starvation.

## Discussion

**FIGURE 8:**
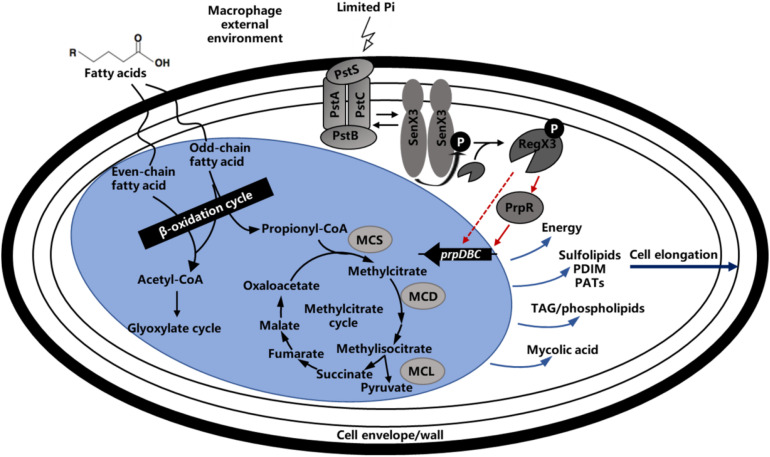
Regulatory model of methylcitrate pathway by RegX3 in *Mycobacteria*. Phosphate-responsive RegX3 and propionate-responsive PrpR competitively regulate the transcription of *prpDBC* operon involved in methylcitrate pathway (MCS, MCD, and MCL), which play an important role in metabolism of cholesterol and fatty acids as the main nutrient sources of mycobacteria during infection. It was suggested that RegX3 contributed to establish a direct association between phosphate and carbon metabolisms, and also modulate the C-P balance in mycobacteria. The wave lines indicate transmembrane transport. The fold line with arrow indicates signal stimulus. The red lines with arrows indicate positive transcriptional regulation while the light blue lines with arrows indicate compound synthesis process.

It has been previously reported that *M. tuberculosis* utilizes fatty acids and cholesterol as main energy source during the course of infection. In *M. tuberculosis*-infected alveolar macrophages, the long-chain fatty acids such as dipalmitoyl phosphatidylcholine from the lung surfactant can be metabolized by *M. tuberculosis* through the glyoxylate cycle (Grabner and Meerbach, 1991; Munoz-Elias and McKinney, 2005). In addition, *M. tuberculosis* can not only hydrolyze the lipids in phagosomal membrane and release free fatty acids (Kondo et al., 1985), but also utilize the macrophage triacylglycerol stores motivated during phagocytosis (Mason et al., 1972). Therefore, fatty acids metabolism plays an important role in *M. tuberculosis* survival within host cells. When lipids act as the primary nutrient of strain, the metabolites acetyl-CoA and propionyl-CoA were degraded by glyoxylate and methylcitrate cycles. It is worth mentioning that the production of propionyl-CoA could be degraded by other way like citrate and methylmalonyl-CoA routes, but the studies showed that methylcitrate rounds is the primary route (Textor et al., 1997; Horswill and Escalante-Semerena, 1999; Claes et al., 2002). As illustrated in Figure 8, when propionyl-CoA enters into the methylcitrate cycle, it would react with the oxaloacetate from TCA cycle to generate methylcitrate under the function of methylcitrate synthase (MCS). With the assist of methylcitrate dehydratase (MCD), the methylcitrate would subsequently be converted into methylisocitrate. And methylisocitrate lyase (MCL) would catalyze the cleavage of methylisocitrate to free pyruvate and the succinate which would reflow to TCA cycle. All homologs enzymes of methylcitrate cycle were encoded in the *M. tuberculosis*, except for MCL, which is characterized by ICL1 and ICL2 enzymes essential for the for survival of *M. tuberculosis* on propionate (Cole et al., 1998). In the *M. smegmatis* genome, a dedicated MCL, which is arranged in the *prpDBC* operon encoding MCS and MCD orthologs, can be potentially encoded (Upton and McKinney, 2007). In addition, the similar arranged orthologs of *prpDBC* are present in the *Mycobacterium marinum* and *Mycobacterium avium* genomes (Li et al., 2005). In summary, the methylcitrate cycle of *M. smegmatis* is generally similar to that of *M. tuberculosis*, it is suspected that these two genetically related mycobacteria share the way of propionate metabolism.

In this work, we showed that the important nutrient-sensing regulator of *M. smegmatis*, RegX3, directly activated the transcription of *prpR* gene involved in methylcitrate cycle in response to phosphate starvation. It was suggested that RegX3 contributed to establish a direct connection between P and C metabolisms, and also modulate the C-P balance in mycobacteria. Carbon and phosphorus are the basic components of microbial nutrition. Pathogenic bacteria may frequently encounter limited supplies of phosphate after ingested by macrophage, although the concentration of soluble phosphate in macrophage has not been determined yet. The regulation of propionate utilization in *M. smegmatis* is under RegX3 control, indicating that phosphate limitation triggers the RegX3-mediated response, which results in the expression of genes involved in propionate degradation and utilization. It was previously reported that PrpR exerts a regulatory effect on the uptake and utilization of propionate in *M. tuberculosis* and influences the expression of MCD and MCS enzymes in methylcitrate pathway by directly regulating the transcription of the local *prpDC* target (Masiewicz et al., 2012). Meanwhile, the *prpDBC* (encode MCD, MCL, and MCS, respectively) operon could also be upregulated by PrpR in *M. smegmatis*. Therefore, we suspected that the increased expression of RegX3, which responded to phosphate restriction, could activate the transcription levels of *prpBCD* genes by its direct regulation of *prpR*, to promote the whole methylcitrate pathway. We found that the phosphate limitation and overexpression of *regX3* increased the activity of MCS, thereby enhancing the propionyl-CoA assimilation (Figures 3B, 4B). Besides, RegX3 was previously shown to regulate the methylcitrate cycle by activating *prpC* expression in *M. tuberculosis* (Roberts et al., 2011). These collective results suggested that the phosphate regulator RegX3 functions at two different levels (*prpR* and *prpC*) in the methylcitrate cycle, thus playing a dual role in both carbon and phosphate metabolism in mycobacteria. More importantly, our data clearly demonstrated that the RegX3-mediated regulatory effect on methylcitrate pathway is conserved in *M. tuberculosis*, which provides insights into the molecular mechanisms for nutrient sensing and metabolism in *M. tuberculosis*. These mechanisms might be general in mycobacteria for nutrient sensing and environmental adaptation.

Transcription inhibition of the *regX3* gene resulted in growth defect on propionate as the sole carbon source. The macrophage infection experiment confirmed that RegX3 is required for the proliferation and survival of *M. smegmatis* in macrophages. How RegX3 enables bacteria to quickly adapt to the harsh macrophage microenvironment and keep infective activity for a long term? On one hand, actinomycetes (including mycobacteria) accumulate large amount of triacylglycerols in response to the starvation condition (Alvarez and Steinbuchel, 2002), so RegX3 stimulated by low-phosphorus signal might accelerate the methylcitrate cycle to prevent the accumulation of toxic metabolites caused by incomplete propionyl-CoA metabolism. On the other hand, it has been discovered that *M. tuberculosis* undergoes elongation following phosphate depletion and the SenX3-RegX3 regulatory system has a great effect on the bacterial morphology (Rifat et al., 2009; Rifat and Karakousis, 2014). The similar cases were also observed when the *M. smegmatis* was cultured on propionate as the main carbon source. There is a reasonable interpretation that RegX3 can elongate bacteria and increase the ratio of surface area to volume so that the bacteria can be more exposed to the surrounding environment to increase the chance of obtaining propionate and other nutrients. Therefore, the RegX3-mediated C-P balance in mycobacteria probably mentions the effect of RegX3 on cell wall or relevant precursor synthesis pathway, which means that the *M. tuberculosis* inside host may control the metabolic level by sensing the change of environmental phosphorus concentration, altering the cell morphology for a stable nutritional status. Furthermore, *M. tuberculosis* cell wall contains various lipid components that can serve as virulence factors, regulate host immune response, counteract anti-TB antibiotics and toxic molecules derived from host cells, it is thus suggested that RegX3 might have some connection with the pathogen-associated molecular patterns (PAMPs) of *M. tuberculosis* (Siegrist and Bertozzi, 2014). It is quite possible for RegX3 to be involved in the interaction between *M. tuberculosis* lipid metabolism and hots immune response, representing a complex and dynamic host-pathogen interplay (Figure 8).

Here, we carried out the research about the regulation mode of transcription regulator RegX3 on methylcitrate cycle in *M. smegmatis.* The experimental results verified by this work could match with the corresponding regulatory network of *M. tuberculosis* to a certain extent, thus reflecting the following metabolism of fatty acid intermediate metabolites in *M. tuberculosis* when the bacteria infect lung cells or survive in macrophages. It is the most important that the research can help us to comprehend how *M. tuberculosis* survives in a harsh host environment, thus extending our understanding of the toxicity mechanism of *M. tuberculosis* and providing new insights into the development of anti-tuberculosis drug.

## Materials and Methods

### Bacterial Strains, Plasmids, and Growth Conditions

Different strains, plasmids referenced in the work are showed in Table 1. *E.coli* used for cloning and protein expression were grown in liquid or onto solid (with the addition of 15 g agar l^–1^) Luria Broth (LB) medium. Different *M. smegmatis* strains, including wild-type *M. smegmatis* MC^2^ 155 (Munoz-Elias and McKinney, 2005), *regX3* knock-down strain and *regX3* overexpression strain, were grown on LB media with 0.05% Tween-80 for strain activation. Phosphate restrictive media were prepared using a MOPS-propionate basal media (5.232 g MOPS, pH 7.2, 1.864 g KCl, 0.584 g NaCl, 1.321 g (NH_4_)_2_SO_4_, 0.017 g FeCl_3_, 0.241 g MgSO_4_, 0.011 g CaCl_2_, 0.961 g Sodium propionate, 0.02% tyloxapol) supplemented with NaH_2_PO_4_ as Pi source. The growth of *M. smegmatis* strains was investigated in the media MOPS-glucose (10 mM) minimal medium with low concentration phosphate (100 μM K_2_HPO_4_) and MOPS-propionate (10 mM) minimal medium with high concentration phosphate (10 mM K_2_HPO_4_). The medium were cultured at 37°C with agitation of 220 rpm and sterilized at 121°C for 20 min.

**TABLE 1:** Strains and plasmids used in this study.

Strains/plasmids	Relevant characteristics	References
**Strains**		
*Mycobacterium smegmatis* MC^2^ 155	Wild-type strain	Jenkins et al., 2012
*Mycobacterium smegmatis* i *regX3*	*regX3* transcription inhibition strain; gentamycin resistant	This study
*Mycobacterium smegmatis* OE *regX3*	*regX3* overexpression strain carrying pMV261-*regX3*	This study
*Escherichia coli*		
DH5α	Recipient for cloning experiments	Novagen
BL21(DE3)	F^–^ *ompT hsdS gal dcm* (DE3)	Novagen
**Plasmids**		
pET28a(+)	Expression vector; Kan^*r*^	Novagen
pET28a-*regX3*	pET28a derivative carrying *regX3* of *M. smegmatis*	This study
pET28a-*prpR*	pET28a derivative carrying *prpR* of *M. smegmatis*	This study
pPR27	*Escherichia coli*- *M. smegmatis* integrative shuttle vector; gentamycin resistant	Yao et al., 2016
pPR27-hsp60-dCas9-*sgRNA*	*regX3* transcription inhibition plasmid; gentamycin resistant	This study
pMV261	Escherichia coli- M. smegmatis integrative shuttle vector containing a strong constitutive hsp60 promoter; kanamycin resistant	Yao and Ye, 2016
pMV261-*regX3*	pMV261 carrying an extra *regX3* for gene overexpression	This study

### Overexpression and Purification of His6-RegX3 and His-PrpR Protein in *E. coli*

The genes (*regX3, prpR, senX3*, and *regX3-Mtb*) were amplified from *M. smegmatis* MC^2^ 155 (Munoz-Elias and McKinney, 2005) and *M. tuberculosis* genome by PCR using a couple of primers (Table 2). The fragments were obtained through digestion of the PCR products using the *Eco*RI and *Hin*dIII for *regX3* and *prpR*, *Nde*I and *Xho*I for *senX3* and *regX3-Mtb*, and inserted into pET-28a(+) vector. The pET-28a(+)-*regX3* plasmid (p0937), pET-28a(+)-*prpR* plasmid (p6643), pET-28a(+)-*senX3* plasmid (p0936), or pET-28a(+)-*regX3-mtb* plasmid (p0467) were transferred to BL21 *E. coli*. *E. coli* strains newly transferred with p0937/p6643/p0936/p0467 were cultured in LB media at 37°C with agitation of 220 rpm (OD_600_ = 0.6). The following overexpression and purification of RegX3, PrpR, SenX3, and RegX3-mtb in BL21 were operated as previously described (Yao and Ye, 2016). The expression of proteins were analyzed by SDS-PAGE, and the concentration was quantified using the Bradford method (Transgen).

**TABLE 2:** The oligonucleotides used in this study.

Oligonucleotides	Sequence (5′ to 3′)	Purpose
**Primers for *M. smegmatis* recombinant RegX3 homolog overexpression**		
*regX3*^Msm^ -F(MSMEG_0937)	CCG*GAATTC*ATGATGTTGATCGTTGAAGACGAGG	forward primer for RegX3 expression
*regX3*^Msm^ -R(MSMEG_0937)	CCC*AAGCTT*TCAGCCCTCCAGCTTGTATCC	reverse primer for RegX3 expression
**Primers for *M. smegmatis* recombinant SenX3 homolog overexpression**		
*senX3*^Msm^	GGCCATATGGTGTCGCTCCTCACGCTG	forward primer for SenX3 expression
*senX3*^Msm^	CCGCTCGAGTCATCGTTCTCGTTGGTCCTC	reverse primer for SenX3 expression
**Primers for *M. smegmatis* recombinant PrpR homolog overexpression**		
*prpR*^Msm^ -F(MSMEG_6643)	ATGGGTCGCGGATCC*GAATTC*GTGGCGAAGACGTTCATAGGCC	forward primer for PrpR expression
*prpR*^Msm^ -R(MSMEG_6643)	CTCGAGTGCGGCCGC*AAGCTT*TCAGCGGGGGTCGTTGACC	reverse primer for PrpR expression
**Primers for *M. tuberculosis* recombinant RegX3 homolog overexpression**		
*regX3*^Mtb^	CCGCATATGATGTCTGTCGTCGGCACC	forward primer for Mtb-RegX3 expression
*regX3*^Mtb^	GGCCTCGAGCTAGTGGAACTGGCCCTCTTC	reverse primer for Mtb-RegX3 expression
**Primers for *M. tuberculosis regX3* knock-down strain**		
Primers for *regX3* knock-down		
hsp60-F	AGCTCCACCGCGGTG*GCGGCCGC*CGGTGACCACAACGACGC	Hsp-60 strong promoter forward primer
hsp60-R	CGTCCTTGTAGTCCATCATATGTGCGAAGTGATTCCTCCGG	Hsp-60 strong promoter reverse primer
dCas9-F	CCGGAGGAATCACTTCGCACATATGATGGACAAGAAGTACAGCATC	dCas9 protein forward primer
dCas9-R	CGGGGGATCCACTAGT*TCTAGA*TCAGTCGCCGCCGAGC	dCas9 protein reverse primer
sgRNA-F	GCTCGGCGGCGACTGA*TCTAGA*GGAAACAGCTATGACCATGATTACG	sgRNA forward primer
sgRNA-R	GAGGATCGGGGGATCC*ACTAGT*AAAAAAAGCACCGACTCGGTG	sgRNA reverse primer
**Primers for construction of the *M. smegmatis regX3* overexpression strain**		
O*regX3*-F	CCGGAATTCATGCATCATCATCATCATCATATGTTGATCGTTGAAGACGAGG	forward primer of regX3 with *Eco*RI Enzyme loci
O*regX3*-R	CCC*AAGCTT*TCAATGATGATGATGATGATGGCCCTCCAGCTTGTATCC	reverse primer of regX3 with *Hin*dIII Enzyme loci
**Primers for PCR amplification of EMSA probe with biotin labeling**		
Universal primer	Biotin-AGCCAGTGGCGATAAG	
*M. smegmatis*		
E6643F	AGCCAGTGGCGATAAGCCGACGGGTGACCGAGGC	forward primer of prpR with 5’-Biotin
E6643R	AGCCAGTGGCGATAAGGCTCTTCCCGCAACCTGCG	reverse primer of prpR with 5’-Biotin
E6643-1F	AGCCAGTGGCGATAAGCCGACGGGTGACCGAGGC	forward primer of prpR with 5’-Biotin
E6643-1R	AGCCAGTGGCGATAAGCGCCGCCGACCCGGTC	reverse primer of prpR with 5’-Biotin
E6643-2F	AGCCAGTGGCGATAAGACCTGCGCGATCTTCCACGCG	forward primer of prpR with 5’-Biotin
E6643-2R	AGCCAGTGGCGATAAGTGCGTAGCGTGCGGATCATGCG	reverse primer of prpR with 5’-Biotin
E6643-3F	AGCCAGTGGCGATAAGGCCCCTAATGGCACGTAAAGAGCCTGTATATGCG	forward primer of prpR with 5’-Biotin
E6643-3R	AGCCAGTGGCGATAAGACACACAGCCTAACCGCGATTCTTACACAGGATTTG	rorward primer of prpR with 5’-Biotin
EM6643-3F	AGCCAGTGGCGATAAGGCCCCTAATGGCACGTAAAGAGCCTGTATATGC	forward primer of prpR with 5’-Biotin
EM6643-3R	AGCCAGTGGCGATAAGACACACATCCTCACCGCGAGGATTACACAGG	reverse primerof prpR with 5’-Biotin
*M. tuberculosis*		
E1129cF	AGCCAGTGGCGATAAGGCACCGGATGCGCCAGT	forward primer of regX3 with 5’-Biotin
E1129cR	AGCCAGTGGCGATAAGCGGTAAGACATTACTCCGCGTCAT	reverse primer of regX3 with 5’-Biotin
**Primers for quantitative RT-PCR (qPCR), *M. smegmatis***		
RT0937-F	TCCCTTGGCGTTTCTGCTC	RT-PCR forward primer of regX3
RT0937-R	CCTTGTCGATCTCGCTGTCG	RT-PCR reverse primer of regX3
RT6643-F	GCCGAGGAGTTGCGCTATG	RT-PCR forward primer of prpR
RT6643-R	TTGGGCGAATGCCTGGTG	RT-PCR reverse primer of prpR
RT6645-F	CACCTGACGACGGCCACC	RT-PCR forward primer of prpD
RT6645-R	GCGGAATGCTCCTTGGTGTAG	RT-PCR reverse primer of prpD
RT6646-F	ATGAGCGCTGCCCGGACC	RT-PCR forward primer of prpB
RT6646-R	GGCATATGTCTTGGCCCTTTCG	RT-PCR reverse primer of prpB
RT6647-F	TTGACCCCGATCCCTCCGCAC	RT-PCR forward primer of prpC
RT6647-R	CCTTCTCCTTGCGGGATAGTTTGCC	RT-PCR forward primer of prpC
sgRNA	GGAAACAGCTATGACCATGATTACGCCAAGCTTGCATACCCGCAGCCCGGGCACCA ATTTGTGATTAGGGCTTGACGGCCGCCCGGCCAGTAGTACATCCTTGTGTCACCG CAACAACAGAGCGGTAGGCGGATCCGAGGGGATCGTGCCGGTGCCGGCGAGGACC CGGCGGCGGGACTCCCCGGTCTGACAGCCACCCGGTCATTGGGTAAGCTGCGGGC ATCACCAACTTGGACGGGAAAGGGAGATCGCATGCGTTTCTGCTCCGTAAGGATTTTA GAGCTAGAAATAGCAAGTTAAAATAAGGCTAGTCCGTTATCAACTTGAAAAAGTGGCA CCGAGTCGGTGCTTTTTTT	

*Italic letters indicate the restriction enzyme sites. The sequences with underlining are universal primers used for biotin labeling.*

### Electrophoretic Mobility Shift Assays

The upstream regions from -300 bp to 0 bp of detected genes (predicted to contain a RegX3-binding site) were amplified by PCR using gene-specific primers and the biotin-labeled universal primer. The probes were confirmed by 1.5% agarose gel and cleaned up using the PCR Purification Kit (Qiagen), whose concentration was detected with microplate reader (Biotek, United States). EMSAs were processed on the basis of the protocol attached to Chemiluminescent EMSA Kit (Beyotime Biotechnology, China). The binding system, containing 1.576 g Tris-HCl (pH = 8.0), 2.38 g Magnesium chloride, 2.92 g Sodium chloride, 1 mM DTT, 0.292 g Ethylene Diamine Tetraacetic Acid, 0.01% Non-idet P40, 50 μg ml^–1^ poly[d(I-C)] and 100 ml glycerol. The reaction system was prepared that probes labeled with biotin cultured separately with different regulator protein at room temperature (20 min) prior to separating on a 6% non-denaturing PAGE gel in ice-bathed TBE at 170 V, and bands were checked by BeyoECL Plus. Sequences of primers are listed in Table 2.

### Construction of the *RegX3* Knock-Down and Overexpression Strains

The *regX3* gene overexpression mutants were generated by protoplast transformation with *E. coli*–*M. smegmatis* integrative shuttle vector pMV261 containing a strong constitutive *hsp60* promoter by inserting the 678 bp of the *regX3* open reading frame (MSMEG_0937, amplified with primers OregX3-F/R) between *Eco*RI and *Hin*dIII, to obtain the *regX3* (pMV261-*regX3*) overexpression plasmid (Table 1; Stover et al., 1991). The plasmids were entered into the *M. smegmatis* WT strain protoplasts by electrotransformation. Kanamycin-resistant was checked by supplemented with 25 μg/ml Kanamycin (final concentration) after transfer for 3∼4 h. The selected recombinant strains of above two kinds of *M. smegmatis* were verified by their relative transcription levels with WT strain via the combination of RNA extraction and real-time RT-PCR. The fold change of *regX3* expression between the overexpression strain and wild-type in rich and nutrient-limiting media were 1.2-fold and 2.5-fold (*p* < 0.05), respectively.

The CRISPRi technique (Yao et al., 2016) was used to construct a mutant with inhibited transcription level of *regX3*. First, we assembled the functional plasmid pPR27-hsp60-dCas9-sgRNA, where the pPR27 plasmid is a shuttle plasmid (Bigi et al., 2004) and the hsp60 is a strong constitutive promoter for *M. smegmatis*, while the complex consisted of the deactivated Cas9 (dCas9) and the sgRNA could bind to target gene and impede the occurrence of transcription. Second, the recombinant plasmids were electroporated into the *M. smegmatis* WT strain protoplasts and the strains successfully introduced plasmid were screened out with gentamycin at a final concentration of 20 μg ⋅ ml^–1^. The *regX3* transcript fold change of the mutants were also verified by comparing with WT strain.

All the primers used for construction of the *M. smegmatis* mutants are listed in Table 2. The designed sgRNA-*regX3* forward sequence (in Table 2) was synthesized by Sangon Biotech (Shanghai) Co., Ltd.

### RNA Preparation and Real-Time RT-PCR

Frozen bacterial stocks were inoculated into LB-medium supplemented with 0.05% Tween 80 and cultured to OD600 of 0.6. Cultures were adjusted to OD_600_ = 0.2 in MOPS-Pi and OD600 measurements were taken every 12-h for 3 days. IE regX3 strains including the pPR27 plasmid were cultured in medium with 50 mg/ml kanamycin (Sigma). overexpression strain including the pMV261 plasmid were cultured in with 50 mg/ml gentamycin (Sigma). For RNA preparation, *M. smegmatis* WT, i *regX3* and OE *rgX3* strains were precultured in LB + 0.05% Tween-80 broth to an OD_600_ of 0.6-0.8 and subsequently transferred to the MOPS-propionate (final OD_600_ 0.2) minimal medium described above with 100 μM K_2_HPO_4_ as unique phosphate resource for several-hour incubation at 37°C after washed three times with sterile physiological saline. Cell pellets were harvested after 10min of centrifugation at 6,000 rpm and 4°C accompanying three-time washing with phosphate buffer saline (PBS). Total RNA was extracted using RNAprep Pure Cell/Bacteria kit (Tiangen Biotech, Beijing, China), with the RNA integrality tested by 1% agarose gel. The obtained RNA was reverse-transcribed to cDNA using RT reagent kit (Takara, Shiga, Japan) following the manufacturer’s instructions. The SYBR premix Ex Taq^TM^ GC Kit (Takara) was chosen as a reaction reagent and final concentration of cDNA was 50 ng/uL. The PCR was conducted using CFX96 real-time system (Bio-Rad, United States) with the primers listed in Table 2. The RT-qPCR experiment methods were described below: 95°C (3 min), then 40 rounds of 95°C (15 s), 60°C (15 s), and 72°C (30 s), finally 72°C (10 min). The 16S rRNA selected as an reference and fold change were analyzed using the (2^–^^Δ^^Δ^^CT^) method or another (2^–Δ^^*CT*^) method when *sigA* gene was used as an internal reference (Elliott and Tischler, 2016).

### Methylcitrate Synthase (MCS) Assays

Frozen WT strain was inoculated into LB-media with 0.05% Tween 80 and cultured to logarithmic phase (OD600 = 0.6). After incubation in Pi-rich (10 mM) and Pi-limiting (100 μM), the WT strain was harvested (OD6_00_ 0.6) for MCS extraction. The harvested strain was ultrasonic crushing (200 W, 3 s ultraphonic, 10 s interval, repeat for 30 times) at the ratio of strain: extraction buffer 1000: 1. Then samples were centrifuged (8000 rpm, 10 min, 4^*o*^C) and the supernatant was harvested for MCS reaction. MCS activity was analyzed by the free CoA accumulation, which produced by the reaction of propionyl-CoA and oxaloacetate. While the free CoA accumulation was checked by the reaction of CoA with DTNB (Lu et al., 2019). Reactions System containing 11.92 g HEPES pH 8.0, 5.84 g Sodium chloride, 0.584 g Ethylene Diamine Tetraacetic Acid, 0.04 g DTNB, 0.035 mM propionyl-CoA, 0.4 mM oxaloacetate, and the extract 1-6 ug/ul. After system cultured at 30°C for 10 min, the TNB anion were tested at 412 nm, using a microplate reader (BioTek, United States).

### Kinetic Binding Analysis

The binding affinities of His-RegX3 or His-PrpR with the promoter of *prpR* (encoding MCS) were determined using the Octet Macromolecular Interaction Platform (Fortebio, United States). Firstly, the upstream promoter regions of *prpR* with a concentration of 50 ng μl^–1^ was dissolved in loading buffer (2.383 g HEPES, 0.19 g Magnesium chloride, 0.029 g Ethylene Diamine Tetraacetic Acid, 14.91 g Potassium chloride, pH 8.0). Next, the biotin-labeled promoter was immobilized on a streptavidin-coated bioprobe (Fortebio), and then the bioprobe with the *prpR* promoter fragment was inserted into the purified His-RegX3 (1.7, 3.4, 7, and 14 μM) or His-PrpR (0.9, 1.8, 3.5, and 7 μM) of different concentration gradient, dissolved in running buffer (2.383 g HEPES, 0.19 g Magnesium chloride, 0.029 g Ethylene Diamine Tetraacetic Acid, 14.91 g Potassium chloride, 10 μg/ml bovine serum albumin (BSA), 0.02% Tween-20, pH 8.0). After the protein binding curve tended to be stable, the bioprobe was moved into running buffer to initiate the dissociation reaction. Finally, the raw data processing was performed using Octet Data Analysis Software Version 7.0 to calculate the kinetic binding affinities (K_*D*_). The KD values of RegX3 and PrpR were 165 and 53.2 nM, respectively. The KD value demonstrates that the PrpR has a stronger binding capacity than RegX3. The detailed protocol has been described in previous research (Liao et al., 2014).

### *Mycobacterium smegmatis* Survival in THP-1 Cell

Bacterial infection model of macrophages was mainly modified with reference to the previous report (Jiang et al., 2018). Human THP-1 cell lines (Conservation Genetics, CAS Cell Bank) were grown in 1640 media (Hyclone, United States) containing 10% FBS (Gibco, United States), and adjusted to a final total of 2 × 10^6^ cells. For the induction of a macrophage-like state, the cells were exposed to a 12-h treatment with 10 ng/ml polymethyl acrylate (PMA). After the renewal of medium, adherent cells cultured 8-12 h, and then co-incubated with different *M. smegmatis* at OD_600_ of 0.4 for 2 h (MOI = 10). The infected cells were washed three times with RPMI 1640 media to remove free bacteria and then grown in novel RPMI 1640 media (15 μg/ml gentamicin) for 2, 12, 24, 36, and 48 h. *THP-1* cell were washed two times with LB medium before lysed in LB culture with 0.05% SDS (10 min, 37°C). In different time point, the cell lysates were diluted to three appropriate sets of tenfold serial gradients (10^2^, 10^3^, and 10^4^) and portions were uniformly spread on LB agar plates. The CFU counting was performed on the plates which were Incubated at 37°C for 72–120 h.

### Transmission Electron Microscopy

Bacterial morphology was evaluated by electron microscopy studies. The bacterial liquid was dry on grids and stained using 1% phosphotungstate (PTA). The profile was captured with JEOL *JEM-1400 TEM [120 kV].* The data of each bacteria was tested with ImageJ software. The mean of the length of the bacilli for each strain was calculated.

### Statistical Analysis

All data are presented as mean values with error bars indicating standard deviations (±SD) calculated from three independent experiments. Statistical significance between two groups was determined by unpaired two-tailed Student’s *t*-test using GraphPad Prism 7. Differences were significant when *p* < 0.05.

## Importance

*Mycobacterium tuberculosis* is the pathogen of tuberculosis, which causes millions of deaths and infections every year. Despite the importance of host lipids for maintaining *M. tuberculosis* activities, little is known about the regulation mechanism of lipid metabolism. Here we uncover a novel metabolic regulation mechanism mediated by the phosphate regulator RegX3 of *Mycobacterium smegmatis*, a fast-growing counterpart of *M. tuberculosis*. RegX3 regulates the propionate metabolism responding to environmental phosphate concentration, and alters cell morphology to maintain nutritional steady state. Moreover, the regulation mechanism has been preliminarily proved to be general for *M. tuberculosis*, and other Mycobacteria. Our findings shed lights on how *M. tuberculosis* utilizes lipids to sustain life in nutrient-deficient macrophage, and provide insights for exploring anti-tuberculosis drug targets.

## Data Availability Statement

The original data presented in the study are included in the article/Supplementary Material, further inquiries can be directed to the corresponding author/s.

## Author Contributions

J-FP and Y-XL performed the experiments. B-CY conceived the concept, and supervised and discussed the experiments with JW and NQ. J-FP, Y-XL, and B-CY wrote the manuscript. All authors contributed to the article and approved the submitted version.

## Conflict of Interest

The authors declare that the research was conducted in the absence of any commercial or financial relationships that could be construed as a potential conflict of interest.
