# Supplementary material for: RegX3-Mediated Regulation of Methylcitrate Cycle in Mycobacterium smegmatis
Source: Front Microbiol. 2021 Feb 2;12:619387. doi: 10.3389/fmicb.2021.619387 (PMC7884335; doi:10.3389/fmicb.2021.619387)
Supplement: Supplementary file 1 [file Data_Sheet_1.PDF]

Supporting Information

RegX3-mediated regulation of methylcitrate cycle in *Mycobacterium smegmatis*

Jin-Feng Pei, Nan Qi, Yu-Xin Li, Jing Wo, Bang-Ce Ye

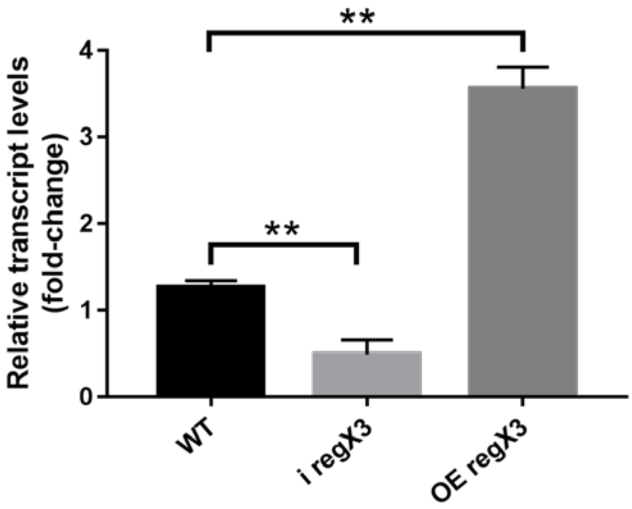

Figure S1. The mRNA levels of *regX3* in *M. smegmatis* wildtype, knock-down and overexpression strains. Wildtype (WT), *regX3* knock-down (i *regX3*) and overexpression strains (OE *regX3*) of *M. tuberculosis* were grown in LB medium, and then harvested at logarithmic phase. RNA was extracted and subjected to qRT-PCR for measuring the transcription levels of *regX3*. Data are presented as means ±SEM calculated from three independent experiments. Unpaired two-tailed Student's t test, $**p<0.01$.

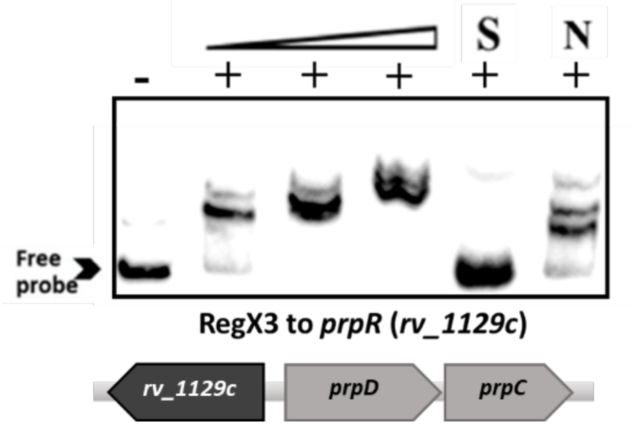

Figure S2. Binding of *M. tuberculosis* RegX3 to the promoter region of *prpR*. EMSA showing the binding of recombinant His-RegX3 with the biotin-labeled DNA probe. The probe was a 300-bp DNA fragment corresponding to the upstream region (-300 to 0 bp of the translational start) of the *M. tuberculosis prpR* gene. Concentration gradient of His-tagged RegX3 (0, 0.5, 1.0, and 1.5 μM) was used. EMSA with a 200-fold excess of unlabeled specific probe (S) or nonspecific competitor DNA (sperm DNA) (N) were conducted as controls. Free probes were indicated by arrowheads. Schematic depiction of the *rv_1129c* (*prpR*) region on the *M. tuberculosis* chromosome was shown.

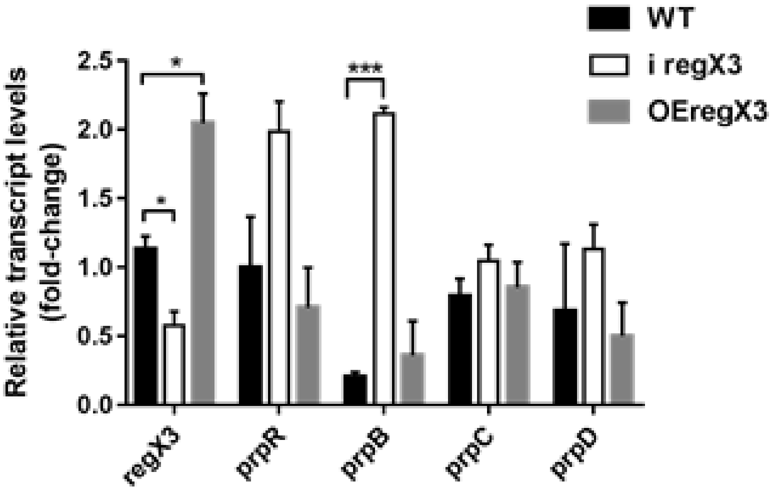

Figure S3. Transcriptional profiles of *regX3* and the methylcitrate cycle-related genes in diverse *M. smegmatis* strains sensing glucose and the low-phosphate condition. WT, i *regX3,* and OE *regX3* strains of *M. smegmatis* were grown in 10 mM glucose minimal medium containing 100 µM K_2HPO_4. As described in Figure 4A, qRT-PCR was performed to measure the relative transcription levels of *regX3*, *prpR*, *prpB*, *prpC* and *prpD*. Data are presented as means ±SEM calculated from three independent experiments. Unpaired two-tailed Student's t test, **p*<0.05; ****p*<0.001.

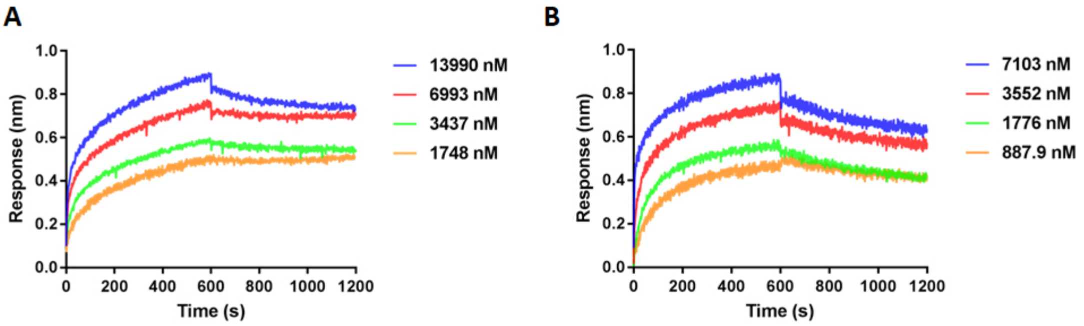

Figure S4. Octet assays of RegX3-*prpR* and PrpR-*prpR*. Binding of *prpR* biotin-labeled probe (1 μg) with indicated concentrations of His-RegX3 (**A**) and His-PrpR (**B**). The KD value of RegX3 and PrpR was 165 nM and 53.2 nM, respectively.

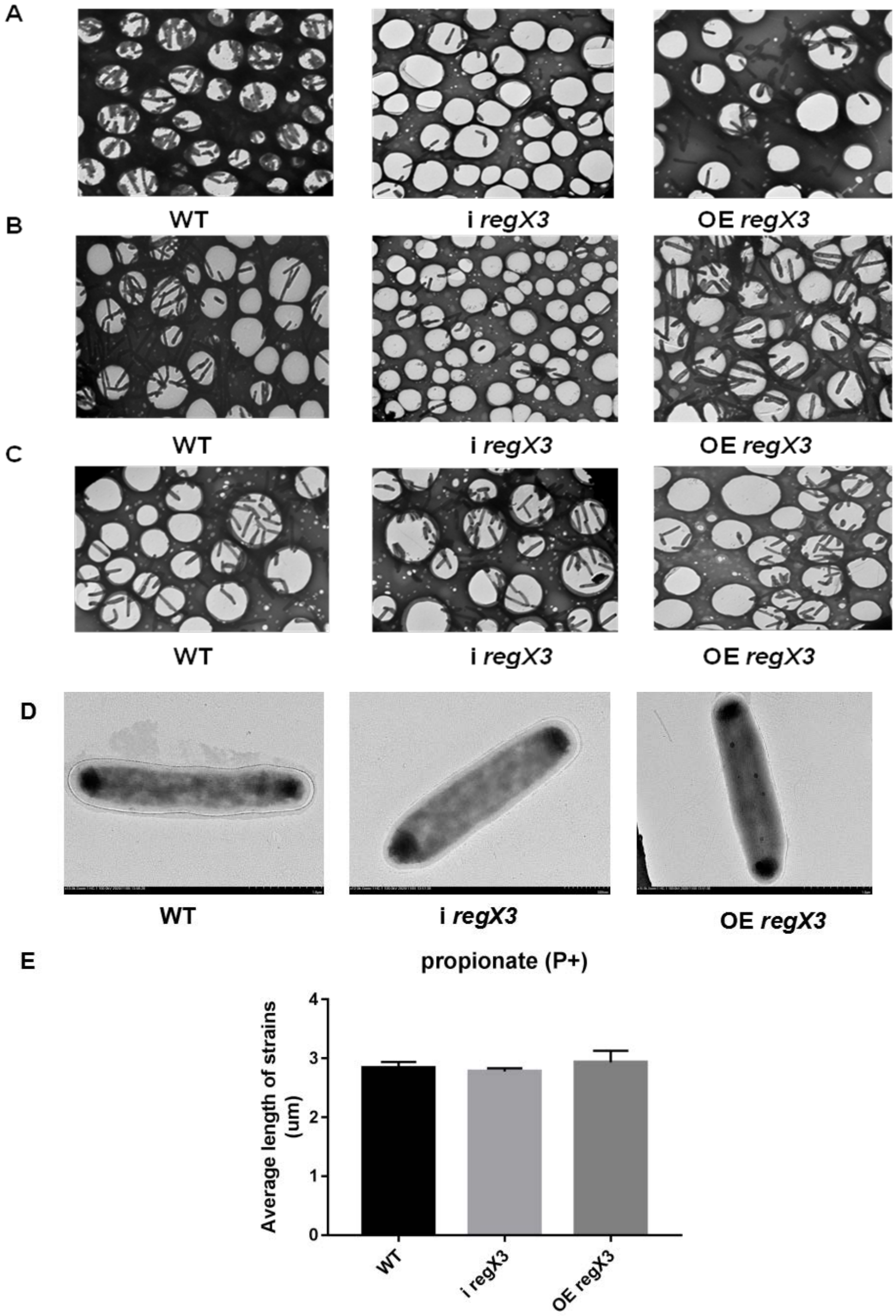

Figure S5. Transmission electron microscopy (TEM) images of bacteria cells from diverse *M. smegmatis* strains in response to different nutrient conditions. Experiment was conducted as described Figure 7. Images of *M. smegmatis* WT, i *regX3,* and OE *regX3* strains grown in (**A**) MOPS-propionate (10 mM) and (**B**) MOPS-glucose (10 mM) medium at a low-phosphate level (P-, 100 μM K_2HPO_4) are related to Figure 7A and 7B, respectively. (**C**) WT, i *regX3,*

and OE *regX3* were cultured in MOPS-propionate (10 mM) at a high-phosphate level (P+, 10 mM K_2HPO_4). (**D**) Representative TEM images of bacteria cells from diverse *M. smegmatis* as described in (C)**. (E)** Length of bacteria cells (n=30) in (C) was measured by manual evaluation, using Image J on the electron microscopic images. Data are presented as means ±SEM.
